# Slowing Alzheimer’s disease progression through probiotic supplementation

**DOI:** 10.3389/fnins.2024.1309075

**Published:** 2024-03-06

**Authors:** Destynie Medeiros, Kristina McMurry, Melissa Pfeiffer, Kayla Newsome, Todd Testerman, Joerg Graf, Adam C. Silver, Paola Sacchetti

**Affiliations:** ^1^Department of Biology, University of Hartford, West Hartford, CT, United States; ^2^Neuroscience Program, Department of Biology, University of Hartford, West Hartford, CT, United States; ^3^Department of Molecular Cellular Biology, UConn, Storrs, CT, United States

**Keywords:** gut microbiota, hippocampus, inflammation, neurodegeneration, neurons, glia, Alzheimer’s disease, entorhinal cortex

## Abstract

The lack of affordable and effective therapeutics against cognitive impairment has promoted research toward alternative approaches to the treatment of neurodegeneration. In recent years, a bidirectional pathway that allows the gut to communicate with the central nervous system has been recognized as the gut-brain axis. Alterations in the gut microbiota, a dynamic population of trillions of microorganisms residing in the gastrointestinal tract, have been implicated in a variety of pathological states, including neurodegenerative disorders such as Alzheimer’s disease (AD). However, probiotic treatment as an affordable and accessible adjuvant therapy for the correction of dysbiosis in AD has not been thoroughly explored. Here, we sought to correct the dysbiosis in an AD mouse model with probiotic supplementation, with the intent of exploring its effects on disease progression. Transgenic 3xTg-AD mice were fed a control or a probiotic diet (*Lactobacillus plantarum KY1032* and *Lactobacillus curvatus HY7601*) for 12  weeks, with the latter leading to a significant increase in the relative abundance of *Bacteroidetes*. Cognitive functions were evaluated via Barnes Maze trials and improvements in memory performance were detected in probiotic-fed AD mice. Neural tissue analysis of the entorhinal cortex and hippocampus of 10-month-old 3xTg-AD mice demonstrated that astrocytic and microglial densities were reduced in AD mice supplemented with a probiotic diet, with changes more pronounced in probiotic-fed female mice. In addition, elevated numbers of neurons in the hippocampus of probiotic-fed 3xTg-AD mice suggested neuroprotection induced by probiotic supplementation. Our results suggest that probiotic supplementation could be effective in delaying or mitigating early stages of neurodegeneration in the 3xTg-AD animal model. It is vital to explore new possibilities for palliative care for neurodegeneration, and probiotic supplementation could provide an inexpensive and easily implemented adjuvant clinical treatment for AD.

## 1 Introduction

Neurodegenerative diseases are slow, progressive, non-reversible conditions that affect neural tissue and eventually result in the loss of cognitive functions. Alzheimer’s disease (AD) is the most widespread form of dementia, which manifests as increasing deficiencies in spatial navigation, memory, and cognition, as well as results in profound changes in personality, including aggressive behavior (Knopman et al., 2021). Neurodegeneration originates in areas involved with memory formation (*e.g.*, the entorhinal cortex and hippocampus) and spreads to cortical areas, affecting acetylcholine neurons and increasingly degrading cognitive functions (Ferreira-Vieira et al., 2016). Neuropathological markers consist of widespread aggregates of amyloid β (Aβ) proteins (plaques) and hyperphosphorylated tau (neurofibrillary tangles), which spread between neurons and induce synaptic losses, cortical atrophy, and increased neuroinflammation (Long and Holtzman, 2019). Aging is a major risk factor for AD, and, as the world’s population ages, the urgency for discovering an effective therapy increases.

Currently, AD treatment via classical pharmaceuticals remains minimally effective and is centered around increasing the synaptic availability of neurotransmitters and targeting Aβ plaques (Breijyeh and Karaman, 2020). However, inflammation is strongly implicated in brain health and neurodegeneration (Guzman-Martinez et al., 2019), and emerging therapies attempt to delay neuronal damage through the suppression of neuroinflammation (Bronzuoli et al., 2016). The inflammatory response of the immune system is vital to fighting infection and repairing tissue damage (Burda and Sofroniew, 2014). In the brain, glial cells such as astrocytes and microglia provide scaffolding and metabolic support to neurons as well as phagocytosis of harmful proteins and invading microorganisms (Aloisi, 2001; Jäkel and Dimou, 2017). Paradoxically, inflammation can also accelerate disease pathogenesis and progression (Liddelow et al., 2017; Sarlus and Heneka, 2017; Kwon and Koh, 2020) and manifests itself in the brain as gliosis, or glial cell activation (Burda and Sofroniew, 2014; Frost and Li, 2017). In a reactive state, astrocytes become hypertrophic and remove Aβ plaques from the intercellular space (Akiyama et al., 2000), but also contribute to Aβ load (Zhao et al., 2011). Upon chronic microglial activation, the cells reorganize into an amoeboid shape, shifting to phagocytic functions and releasing inflammatory signals, which contribute to and exacerbate synaptic pruning and neuroinflammation (Lull and Block, 2010; Serrano-Pozo et al., 2013).

The gut microbiota (GM) consists of a multitude of microorganisms living in the gastrointestinal tract, whose composition depends on a variety of factors, including age, diet, genetics, and environment (Shreiner et al., 2015; Rinninella et al., 2019). Research has shown that the GM is crucial for host health and is intricately linked to host cognition (Carabotti et al., 2015). Interestingly, poor diet and aging contribute to dysbiosis, or the imbalance in the GM, which in turn leads to gut inflammation and enhances systemic inflammation. Low chronic inflammation contributes to neurodegeneration, among other age-related pathologies (Ragonnaud and Biragyn, 2021). Several neurodegenerative diseases are associated with gastrointestinal comorbidities (Vogt et al., 2017; Varesi et al., 2022) and work on animal models of Parkinson’s disease and AD has highlighted the relationship between dysbiosis and neurodegeneration (Sampson et al., 2016; Nair et al., 2018; Sun and Shen, 2018; Doifode et al., 2021). Recent findings linking neural disorders to gut dysbiosis have revealed the gut-microbiota-brain-axis as a potential therapeutic target (Toledo et al., 2022). The term “gut-brain axis” refers to a bidirectional communication pathway between the brain and the gut (Doifode et al., 2021) that depends on bacterial metabolites and neuromodulatory molecules to function (Yano et al., 2015) and involves the neural, endocrine, and immune systems (Kowalski and Mula, 2019; Park and Kim, 2021). For example, microorganisms affect the development and function of neural circuits regulating anxiety (Bravo et al., 2011; Heijtz et al., 2011) and they regulate neurotransmitter production (Yano et al., 2015). In addition, modulation of the GM impacts oxidative stress, oxidative damage (Jones et al., 2012; Dumitrescu et al., 2018), and influences neuroinflammation (Park et al., 2020; Goyal et al., 2021), which are three factors strongly linked to neurodegeneration.

Research suggests that microbial gut dysbiosis could contribute to AD pathology via several mechanisms, such as creating a more permeable gut-epithelial and blood–brain barrier as well as directly and indirectly contributing to systemic and neural inflammation. A weakened gut barrier allows for the passage of bacterial toxins and pathogens into the bloodstream, which leads to systemic inflammation (Zhan et al., 2018). In addition, *Lactobacillus plantarum* MB452 was also shown to enhance the function of the intestinal barrier (Anderson et al., 2010). Gut dysbiosis can also lead to a more permeable blood–brain barrier; for example, altered metabolism of bile acids by gut bacteria was correlated with cognitive impairment as well as linked to the disruption of the blood–brain barrier (Arora et al., 2020). These weakened barriers allow for the translocation of inflammatory agonists to the brain, which leads to the continuous activation of microglia, and subsequent release of proinflammatory cytokines. Probiotics, such as *Lactobacillus plantarum* NCIMB 8826 have been shown to produce ferulic acid (FA), a compound with antioxidative and anti-inflammatory properties (Westfall et al., 2017). Studies have demonstrated that FA reduces free radicals and could potentially reduce the formation, deposition, and maturation of Αβ (Ono et al., 2005). In addition, probiotic supplementation led to an increase in SIRT1 in an AD transgenic mouse model (Bonfili et al., 2018), which has been shown to suppress neural inflammation (Chen et al., 2023).

The relation between microbial dysbiosis and AD has been established (Zhang et al., 2017; Zhuang et al., 2018; Li et al., 2023) and GM modulation has resulted in a reduced load of cerebral Aβ and changes in microglia activation (Minter et al., 2016, 2017; Harach et al., 2017; Dodiya et al., 2019; Kim et al., 2020). However, the direct modulation of the GM as a beneficial therapy in neurodegenerative disorders needs further exploration. Probiotics, prebiotics, postbiotics, antibiotic treatments, and fecal transplants are all effective modalities to alter the GM composition (Ono et al., 2005). The term probiotics refers to beneficial microorganisms that can be safely ingested and provide health benefits for the host (Fooks and Gibson, 2002). It has been shown that interventions with single or multi-strain probiotics can positively impact cognitive and memory deficits in AD animal models (Kobayashi et al., 2017; Nimgampalle and Kuna, 2017; Azm et al., 2018; Bonfili et al., 2018; Lee et al., 2019); but to date, only a limited number of randomized trials have used probiotics as a treatment in AD patients (Naomi et al., 2021) and no cellular mechanism has emerged to explain the beneficial effects.

In the current study, we investigated the effects of probiotic administration on the progression of AD in the 3xTg-AD mouse model harboring transgenes with mutations associated with familial AD, PS1M146V, APPSwe, and TauP301L (Oddo et al., 2003). The probiotics, *Lactobacillus plantarum* KY1032 and *Lactobacillus curvatus* HY7601, used in this study have previously been shown to improve age-dependent memory deficit in rats (Jeong et al., 2015), reduce inflammation in obese mice (Park et al., 2013), and have recently been used in a clinical trial for their anti-obesity effects via gut microbiota modulation (Mo et al., 2022). We hypothesized that the same strains of probiotics would improve memory functions in AD mice possibly via alterations in the gut that could impact neuroinflammation and neuronal loss. We aimed to explore probiotics as a tool to delay tissue degeneration and reinforce their application as an easily accessible adjuvant therapy for AD.

## 2 Materials and methods

### 2.1 Animals

Triple transgenic 3xTg-AD (stock no. 034830-JAX; Jackson Laboratory East, Bar Harbor, Maine) and wild-type B6129SF2/J strain mice (stock no. 101045; Jackson Laboratory East) were used in these experiments. Female mice were housed in groups of 3–4 animals per cage, while males were housed in single cages except for 1 h per day for probiotic treatment when all animals were housed in separate cages to ensure equivalent probiotic dosage for each animal. Access to food and water was *ad libitum* and they were kept on a 12-h light/12-h dark cycle. At the beginning of each experimental iteration, five mice were assigned to each group. For experiment one, wild-type control (WTC), AD control (ADC), and AD probiotic (ADP) consisted of male and female mice aged 3–6.5 months (*n* = 15, average age 4.7 months), while for experiment two, probiotic supplementation of male and female mice began at 7 months of age (*n* = 15). The estimated adequate sample size based on the crude method of degree of freedom of analysis variance for three groups (WTC, ADC, ADP) was 12 and was increased to 15. All experiments were performed in strict accordance with the guidelines described by the National Institute of Health Guide for the Care and Use of Laboratory Animals and the University of Hartford Institutional Animal Care and Use Committee (IACUC).

### 2.2 Probiotic preparation and administration

Probiotic strains *Lactobacillus plantarum* KY1032 and *Lactobacillus curvatus* HY7601 were obtained from Yakult Hansha Co., Ltd., Korea. These strains were originally isolated from kimchi, a traditional Korean fermented cabbage (Chang et al., 2010). Probiotics were preserved at −20°C per direction of the company. Probiotic strain viability was reported in colony forming units (CFU) per gram, and all strain viability data was provided by Yakult Co, LTD. The bacteria were suspended in sterile 10 mM Phosphate-buffered saline (PBS) PBS at a concentration of 5 × 10^10^ CFU/ml for each strain. For each probiotic treatment, individual mice were administered a dose of 10^10^ CFU of *Lactobacillus plantarum* KY1032 and 10^10^ CFU of *Lactobacillus curvatus* HY7601, for a total of 2 × 10^10^ CFU of probiotics per day.

Prior to the first day of probiotic administration, mice across all groups were habituated to the conditions of probiotic administration and trained to ingest a 0.75 g portion of Vanilla Wafer cookie. Probiotic supplementation was provided daily for the entire 12 weeks of the study to the ADP group on a vanilla wafer, while the control groups (ADC, WTC) were provided a vanilla wafer with PBS. To ensure every mouse received and consumed the complete dose, mice were separated temporarily into individual cages until the entire wafer was consumed, which was confirmed visually and in each case the wafer was consumed within 2 min.

### 2.3 Barnes maze experiments

The Barnes Maze (BM) experiments consisted of a training phase and an experimental phase that started at week 7 of probiotic supplementation. The training phase, which took place 1 week prior to the first recorded maze trial, lasted three consecutive days and comprised of 1 day of habituation and 2 days of training. On day 1, mice were habituated to the BM environment in the absence of intra-maze visual cues or an escape chamber. Each mouse was placed into the center of the maze and allowed to interact with the maze for 2 min. On day 2, intra-maze cues and the escape chamber were added to the paradigm and mice were trained to the location of the escape chamber. During the experimental phase, all mice were tested once weekly and a randomized testing order within each group was generated each week. The maze platform was thoroughly sterilized with 70% ethanol prior to each individual trial to remove olfactory cues from the environment. All behavioral tests were video recorded with ceiling-mounted video camcorder equipment and digitally stored for later analysis.

Total escape latency was determined by timing the mouse’s interaction with the maze until the mouse had placed all four paws into the escape chamber, or until the maximum allotted time of 120 s was reached. To determine the total number of errors, researchers manually counted head pokes into false escape holes until the mouse discovered the true escape hole. A head poke was considered valid if the nose of the mouse physically crossed the barrier of the escape hole. For each weekly BM trial, escape latency averages and average error rate for each group (WTC, ADC, ADP; *n* = 5) were determined to represent group performance per week and group averages for the entire experimental phase were calculated.

### 2.4 Immunohistochemistry

Mice were anesthetized in a glass jar using 1.0 ml of 50% isoflurane on gauze pads and once non-responsive to toe pinch, they were perfused transcardially with 0.9% saline followed by 4% (v/v) paraformaldehyde (PFA) in 0.1 M PBS. Brains were removed, postfixed in 4% PFA for 48 h at 4°C, and then immersed in 30% sucrose supplemented with 1% Sodium Azide. Brains were sectioned in 20 μm coronal sections using a cryostat or 50 μm coronal sections using a Vibratome Series 1000 (TPI) 1,000. Areas spanning −0.82 mm to −4.5 mm Bregma were collected covering the CA1 region of the hippocampus and layers IV and V of the entorhinal cortex.

Sections (*n* = 2–3 per animal per stain) were blocked for 30 min in 1x PBS supplemented with Bovine Serum Albumin (BSA) at room temperature and stained overnight at 4°C using antibodies NeuN (Abcam [1:1000], Cat #EPR12763), GFAP (Immunostar [1:500], Cat #22522) and Iba1 (Wako [1:500], Cat #S-2000). Appropriate secondary antibodies (Alexafluor [1:1000]) were diluted in 1x PBS and applied for 2 h at room temperature. Sections were counterstained with 4′,6-diamidino-2-phenylindole (DAPI), coverslipped and stored at +4°C until analysis.

### 2.5 Tissue data quantification

Tissues were observed using a Zeiss Axiostar Plus microscope and images were captured using QCapture camera software and a QImaging camera with 12-bit depth capability. All image post-processing and analysis was done with ImageJ open-source software. In both experiments, for each region of interest (*n* = 2–3 sections per region) in every animal in each groups (*n* = 5), cells were counted manually (*n* = 3 fields per section) using the multi-point function of ImageJ and were used to determine absolute and relative populations for each region of interest. Technical difficulties during staining procedures resulted in exclusion of the section from the data points.

### 2.6 Microbiome analysis

At two time-points during the experiment (week 0 and week 12), fecal samples were collected from each mouse. Week 0 samples, prior to any probiotic treatment, were used to establish a baseline gut microbiota evaluation for each group. Fecal samples were stored in separate tubes for each mouse and preserved at –80^o^C immediately following collection. DNA was extracted from week 0 and week 12 samples using the DNeasy PowerSoil Pro kit (Qiagen), following the manufacturer’s instructions.

#### 2.6.1 PCR amplification

For each sample, the amplification of the V4 hypervariable region of the 16S rRNA gene was conducted using previously validated primers containing dual-end adapters for indexing (Nelson et al., 2014; Benjamino et al., 2018). The PCR reactions were prepared with a total volume of 83.4 μl, consisting of 3 μl of BSA (New England Biosciences (NEB), Ipswich, MA, USA), 41.7 μl of Phusion 2× Master Mix (NEB), 2.5 μl of a 10 μM primer mix (515F forward and 806R reverse rRNA gene V4 primers with Illumina MiSeq adaptors), and 30 ng of sample DNA. To ensure accuracy, each sample underwent triplicate assays using a Bio-Rad C1000 Touch Thermocycler (Bio-Rad Laboratories Inc., Hercules, CA, USA) with the following cycling parameters: an initial denaturation at 94°C for 3 min, followed by 30 cycles of 45 s at 94°C, 60 s at 50°C, and 90 s at 72°C. A final elongation step of 10 min at 72°C concluded the process.

#### 2.6.2 Library preparation and MiSeq sequencing

The triplicate reactions were combined and analyzed on a QIAxcel system (Qiagen) to confirm the size of the amplified products and measure DNA concentrations. Subsequently, the samples were pooled and purified using a GeneRead Size Selection Kit (Qiagen, Cat # 180514) and then diluted to approximately 4 nM in preparation for loading onto an Illumina MiSeq instrument (Illumina, San Diego, CA, USA). Paired-end sequencing of all libraries was performed using an Illumina 500 cycle MiSeq Reagent Kit v2 (Illumina, Cat # MS-102-2003). Sequences can be found on the NCBI database via accession number PRJNA1061566.

### 2.7 Bioinformatic processing of microbiome data

The Illumina MiSeq generated 16S rRNA V4 gene sequence data, which were demultiplexed using BaseSpace^1^. The paired-end data was then imported into QIIME 2 (version 2018.8^2^) for further analysis (Testerman et al., 2022). The reads were filtered, denoised, and dereplicated using the DADA2 denoise-paired plugin (Callahan et al., 2016). Taxonomic classification and compilation of sequences were performed using the feature-classifier (Bokulich et al., 2018) and taxa^3^ plugins. Taxonomy assignment utilized a pre-trained Naïve Bayes classifier based on the GreenGenes 13_8 99% OTU database, with reads trimmed to include only the region bounded by the 515F/806R primer pair. Read counts for specific taxa of interest were obtained from the barplot visualization in QIIME 2 using the csv download function. The default processing parameters of QIIME 2 were employed throughout the workflow unless otherwise specified. ASV counts, taxonomy information, and a phylogeny were imported into RStudio (version 2022.07.02) (R Core Team, 2017) from QIIME 2 using the qiime2R package (version 0.99.6) (Bisanz, 2018). Subsequently, Phyloseq (version 1.36.0) (McMurdie and Holmes, 2013), ggplot2 (version 3.3.6) (Wickham, 2016), microViz (version 0.7.6) (Barnett, 2021), and ANCOM-BC (analysis of composition of microbiomes with bias correction) (version 1.2.0) (Lin and Peddada, 2020) were utilized for calculating alpha and beta diversity, generating barplot, boxplot, and ordination visualizations. Statistical testing was primarily conducted using the vegan package (version 2.6–2; Oksanen, 2020). For a detailed overview of commands and parameters, please refer to the GitHub repository at https://github.com/joerggraflab/Code-for-Medeiros-2023.

### 2.8 Statistical analysis

All behavioral and tissue statistical analyses and graphs were created using Prism software (GraphPad Software, USA). For the behavioral tests, data are presented as mean ± Standard Deviation (SD) computed at the 95% confidence interval. One-way ANOVAs were performed to compare the differences in escape latencies and error rates between groups followed by Tukey *post hoc* test, when significant differences were found. Escape latency time, reported in seconds (s), were calculated as average escape latency for each group (WTC, ADC, ADP) across all maze trials (*n* = 20). Week 7 escape latency and head poke results were discarded due to technical issues during one of the group trials. The average total number of erroneous attempts, reported as number of head pokes, was calculated for each group (WTC, ADC, ADP) across all maze trials (*n* = 20). Tukey multiple comparisons test was performed when significant differences were found and reported as follows: **p < 0.01, ****p < 0.0001.

For the tissue analysis, data are presented as mean ± standard error of the mean (SEM). The non-parametric tests (Kruskal–Wallis and Mann–Whitney) were performed to measure differences between groups for relative population statistical analysis. For each staining, mean values per section (*n* = 3 fields per section) per animal (*n* = 2–3 sections per animal) were calculated and means were compared between groups (ADC vs. ADP; *n* = 5). When regional specificity and sex were analyzed, all sections in each group fitting the paraments analyzed were pooled and means per group were calculated. When significant differences were found, they were reported as follows: *p < 0.05, **p < 0.01.

For the microbiome data, a Shapiro–Wilk normality test was used to assess the normality of each group’s distribution and a t-test was used to assess significant differences between the three groups (baseline, control, and probiotic). A Bonferroni correction was applied to all *p*-values to account for multiple comparisons.

## 3 Results

In the current study, our objective was to assess the efficacy of probiotics, as an early adjuvant therapy, in inhibiting the progression of neurodegeneration and mild cognitive impairment in AD pathology.

### 3.1 Probiotic supplementation improves memory performance

**Figure 1:**
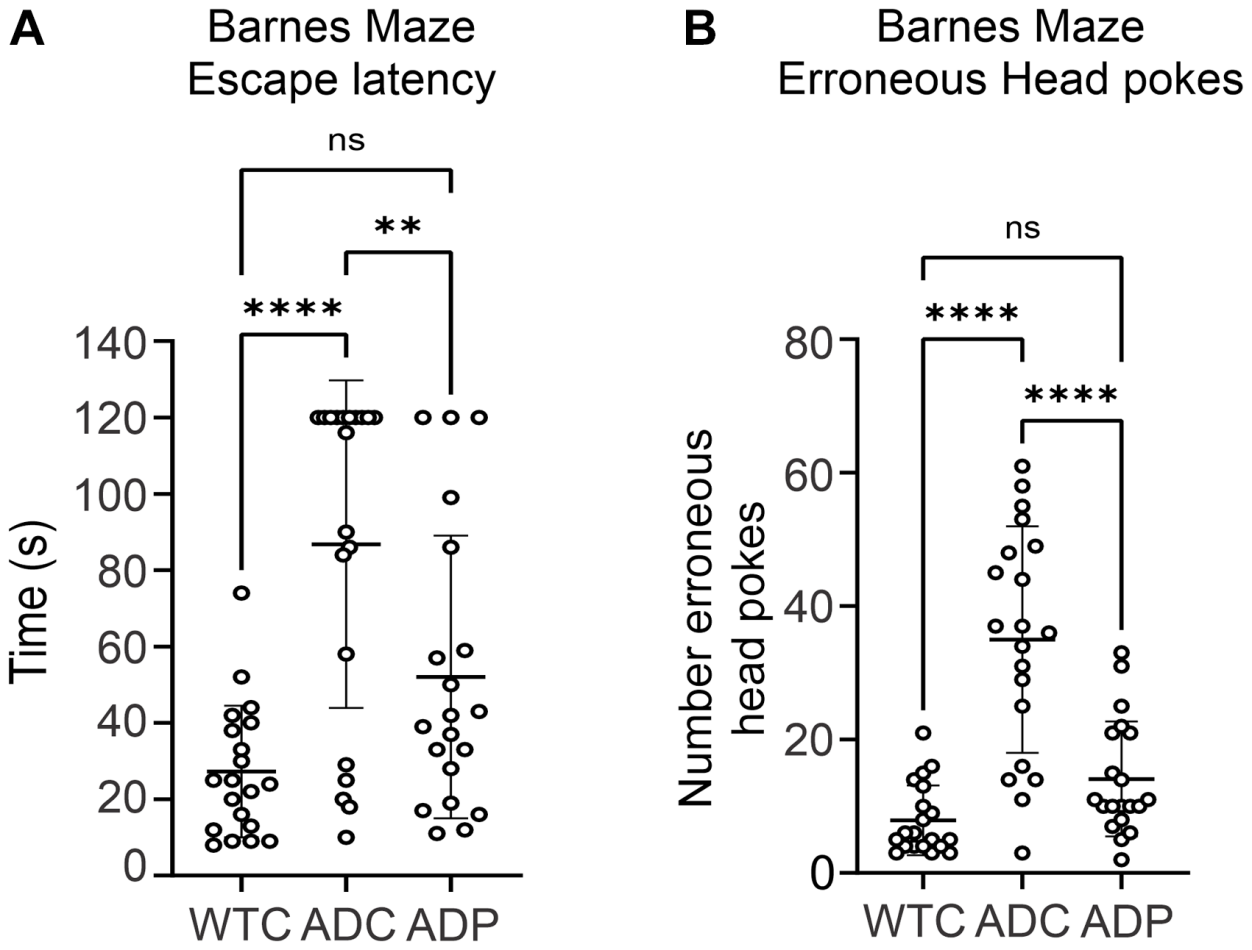
Spatial orientation and memory improved in 3xTg mice treated with probiotics. Each data point represents an individual mouse from a single trial. Each group is represented by 4 weeks of trials for each animal per group (*n* = 5) for escape latency and head pokes, respectively. **(A)** Overall escape latency times represent average escape latency for each group ± SD across all maze trials. Data reported in seconds, as average latency for each group. **(B)** Number of head pokes represent the average total number of erroneous attempts for each group ± SD across all maze trials. Statistically significant differences among groups were analyzed via one-way ANOVA at a 95% confidence interval, using Tukey’s multiple comparisons *post hoc* test. **p < 0.01, ****p < 0.0001.

We evaluated the effects of daily administration of *Lactobacillus curvatus* HY7601 and *Lactobacillus plantarum* KY1032 on spatial learning and memory in a murine Alzheimer’s model, the 3xTg-AD mouse strain (PS1M146VA, PPSwe, TauP301L). AD mice received daily probiotics (ADP) or vehicle (ADC) for 12 weeks and were tested weekly using the Barnes Maze (BM) starting at week seven. Since alterations in microbiome composition can impact behavior even in healthy individuals, only untreated wild-type mice (WTC) were used for comparison in the BM. Throughout these trials, overall escape latency was highest for ADC, indicating poor spatial memory in these mice (Figure 1A). WTC mice displayed the lowest average escape latency time (27.25 ± 17.24 s), followed by ADP mice (52.05 ± 37.05 s), while ADC mice displayed the highest average escape latency of all groups (86.80 ± 42.91 s). There was a significant effect of group on cognitive performance at the *p* < 0.0001 level for the three conditions [*F*(2,57) = 15.29, *p* < 0.0001]. *Post hoc* comparisons using the Tukey HSD test indicated that the WTC group significantly outperformed the ADC group (*p* < 0.0001), but not the ADP group (*p* = 0.0650), while the ADP group demonstrated significantly faster performance compared to ADC (*p* = 0.0060), suggesting a beneficial cognitive effect arising from probiotic treatment.

In addition, ADC mice displayed a greater number of head pokes (35 ± 16.98), which signified high erroneous attempts to escape the maze, compared to WTC (7.90 ± 5.24) and ADP (14.10 ± 8.60) mice (Figure 1B). There was a significant effect of the strain on memory performance (*p* < 0.0001) [*F*(2,57) = 31.03, *p* < 0.0001]. WTC mice displayed significantly lower mean erroneous attempts than ADC (*p* < 0.0001) but not compared to ADP (*p* = 0.2067), while ADP mice displayed significantly fewer erroneous attempts (*p* < 0.0001) compared to ADC mice (Figure 1B).

### 3.2 Probiotic supplementation affects neural populations

To determine if the behavior improvement observed after probiotic treatment was linked to an amelioration of brain milieu, we analyzed the brain tissue in the three groups of mice. Spatial navigation is linked to the entorhinal cortex (EC) and is believed to be the starting point of AD spreading (Igarashi, 2023). Therefore, neuronal and glia populations were evaluated in ADC and ADP mice using immunohistochemistry and compared to WTC mice.

**Figure 2:**
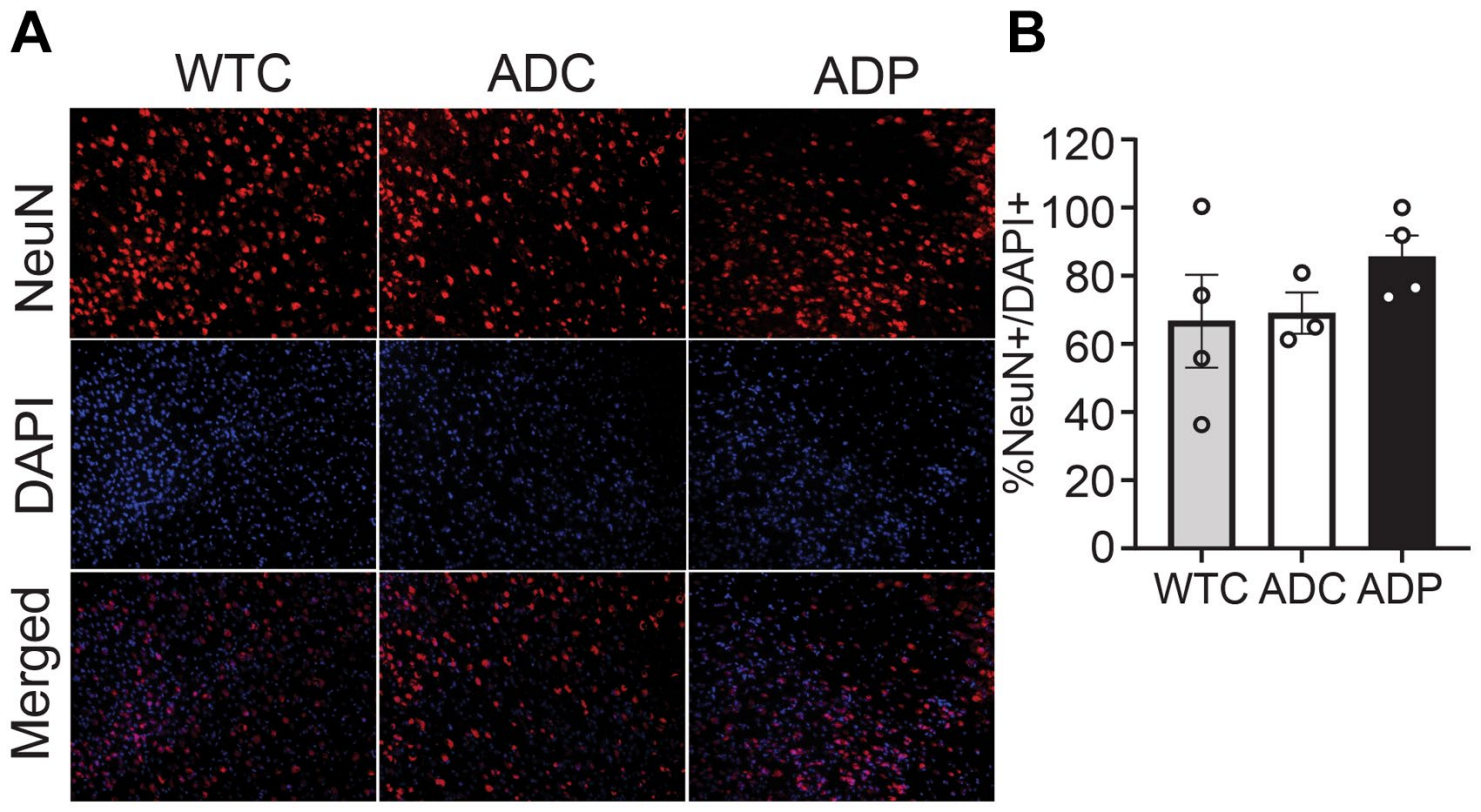
No changes in cortical neuronal populations after treatment with probiotics. **(A)** Representative immunofluorescent staining in layers 4/5 of the entorhinal cortex at 7 months of age, using a NeuN^+^ antibody to tag neurons (top panels) and DAPI nuclear stain to detect total cells (middle panels). Merged images shown in bottom panels. **(B)** In layers 4/5 of the entorhinal cortex, there is no significant difference between the relative number of neurons (NeuN^+^/DAPI^+^) in WTC, ADC and ADP treated mice. Each data point represents the average (*n* = 3 fields per section) percentage of neurons out of total cells per section stained in each group of 5 animals. Data reported as mean percentage for each group ± SEM and analyzed by a Kruskal–Wallis test.

To investigate the neuronal populations, we stained for NeuN^+^ cells and total cells (DAPI^+^) in the EC tissue, specifically in cortical layers 4 and 5 (Figure 2A). The percentage of NeuN^+^ cells (NeuN^+^/DAPI^+^) in the EC did not vary among WTC (66.67 ± 13.65%), ADC (69.04 ± 4.76%), and ADP (85.55 ± 6.25%) mice (Figure 2B).

**Figure 3:**
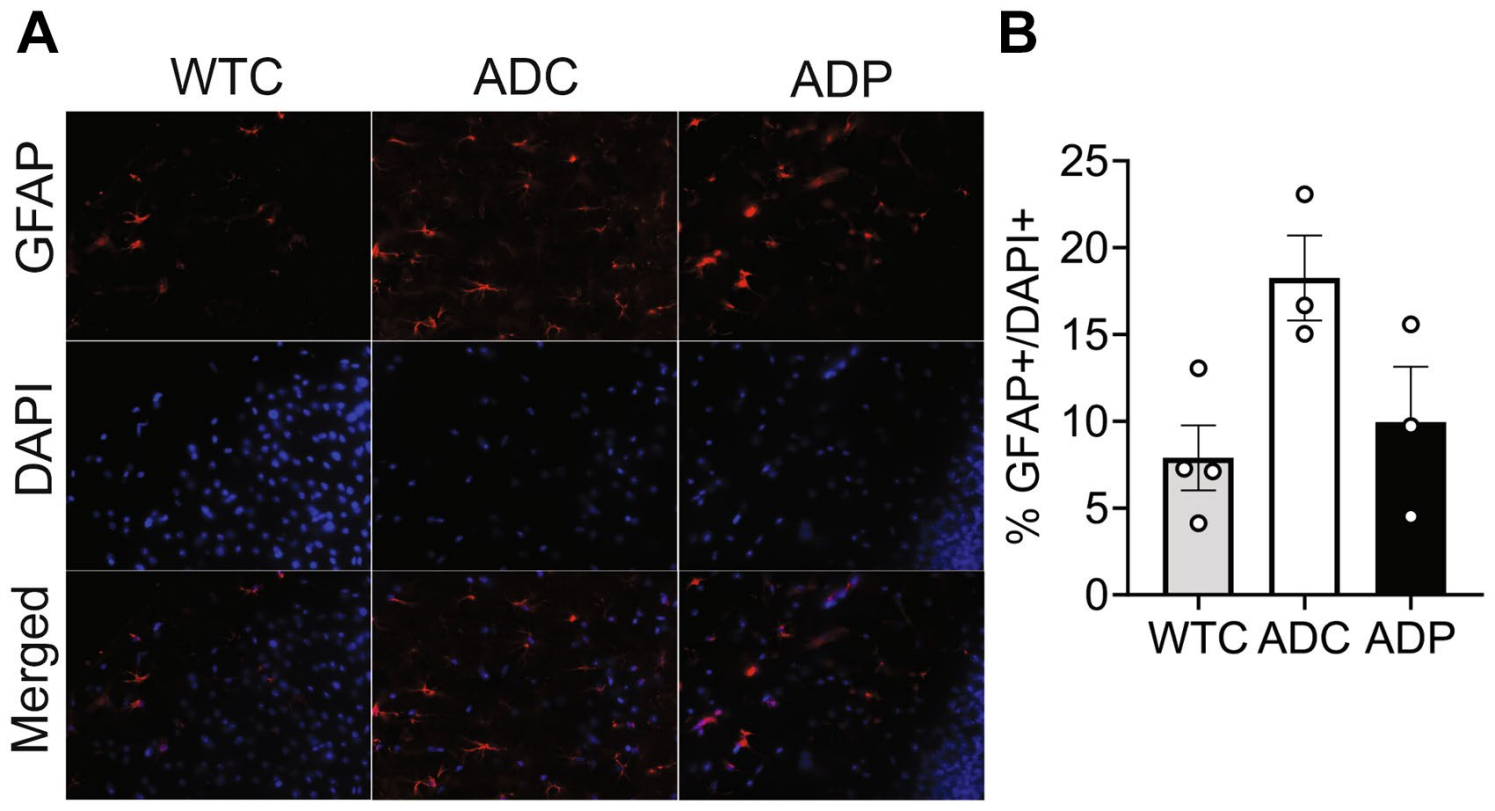
Probiotic treatment affected cortical astrocyte populations in AD mice. **(A)** Representative immunofluorescent staining in layers 4/5 of the entorhinal cortex at 10 months of age using a GFAP antibody to tag astrocytes (top panels) and DAPI nuclear stain to detect total cells (middle panels). Merged images shown in bottom panels. **(B)** Higher percentage of astrocytes were present in the entorhinal cortex of ADC mice compared to WTC, while ADP % astrocytes were comparable to WTC mice. Each data point represents the average (*n* = 3 fields per section) percentage of GFAP out of total cells per section stained in each group of 5 animals. Data reported as mean percentage for each group ± SEM and analyzed by a Kruskal–Wallis test.

Cortical astrocyte populations were investigated by staining for GFAP^+^ cells and total cells in the EC subfields (Figure 3A). The ratio of astrocytes present in the EC was significantly increased in ADC (18.26 ± 4.25%) compared to WTC (7.89 ± 1.87%, *p* = 0.018), which could be indicative of a heightened inflammatory response in AD mice. Interestingly, ADP mice showed a decreased percentage of GFAP^+^ cells (9.94 ± 3.19%) compared to ADC with results comparable to WTC mice (Figure 3B).

**Figure 4:**
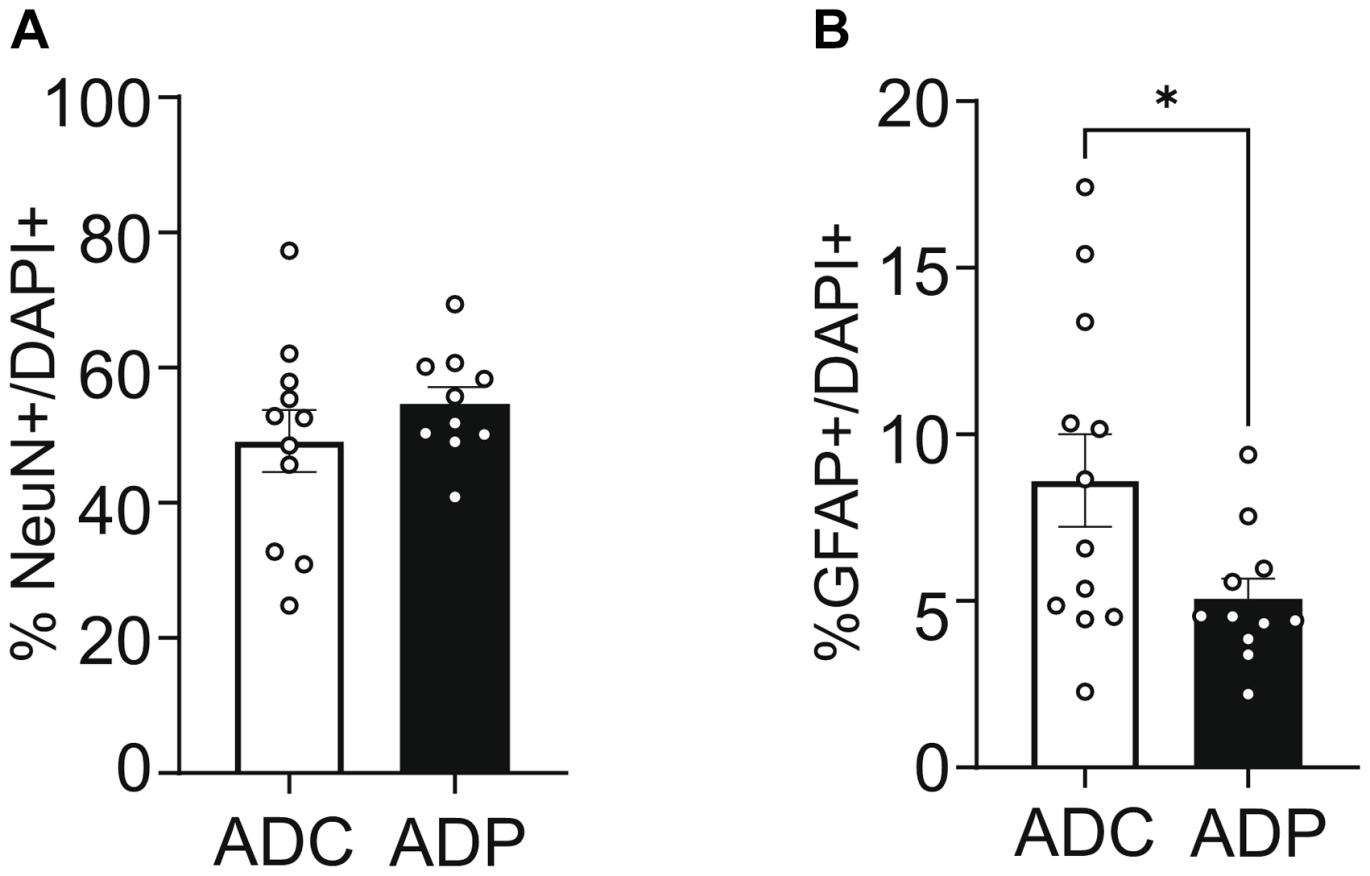
Probiotic treatment altered only cortical astrocytes in AD mice. Each data point represents the average (*n* = 3 fields per section) percentage of neurons or astrocytes out of total cells counted per section stained. For each mouse (*n* = 5 per group), 2–3 sections were stained and pooled per group (*n* = 10–12). **(A)** No changes in cortical neurons (NeuN^+^) were observed in ADC versus ADP, while **(B)** a significant decrease in astrocytes (GFAP^+^) was observed in the entorhinal cortex of 10 months old ADP mice on a 12-week probiotic diet compared to ADC mice. Data reported as mean percentage per group ± SEM and analyzed by a Mann–Whitney test. *p < 0.05.

To further our analysis, we treated a second cohort of 7-month-old AD mice with probiotics or vehicle for 12 weeks and once again examined neural populations in the EC and the CA1 region of the hippocampus. Since our interest lies in probiotics as a tool to slow progression of the disease, we focused our tissue analysis on treated and untreated AD mice only. Analogously to the first cohort, percentages of cortical neurons were similar in ADC and ADP mice (Figure 4A), while cortical astrocytes (GFAP^+^) were decreased in 10-month-old ADP mice compared to ADC (difference 3.07%, *p* = 0.044; Figure 4B).

**Figure 5:**
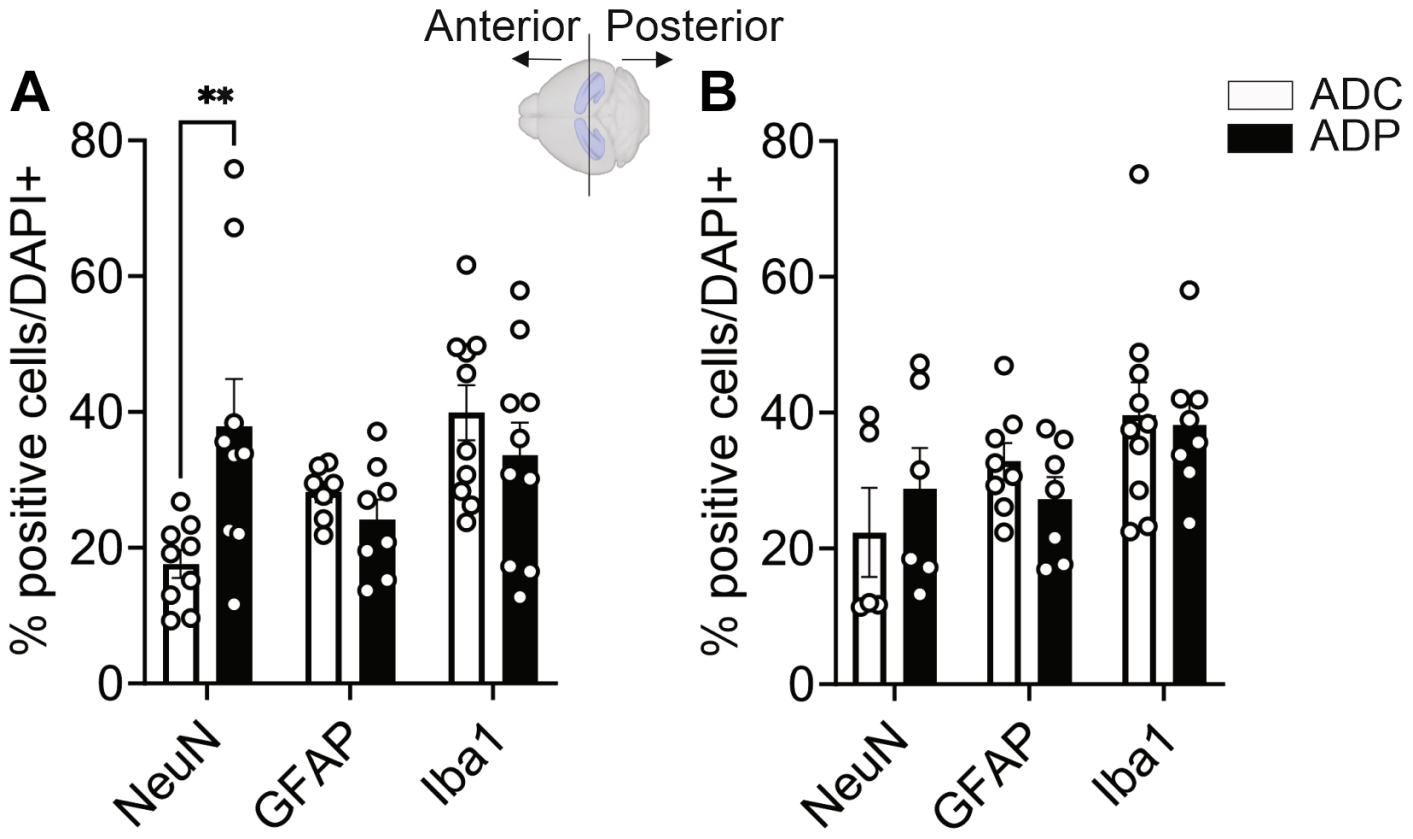
Probiotics treatment protected neuronal cells in the anterior hippocampus in AD mice. Each data point represents the average (*n* = 3 fields per section) percentage of positive cells out of total cells per section stained in each group. For each group, 5 mice were assessed. Based on Bregma location, results were subdivided into anterior vs. posterior area (*n* = 6–10 per area). NeuN^+^, GFAP^+^ and Iba1^+^ cells were counted in the **(A)** anterior and **(B)** posterior hippocampi of ADC and ADP mice. Significant differences were observed in neurons but not glia in anterior hippocampi. Data reported as mean percentage per group ± SEM and analyzed by Mann–Whitney test. **p < 0.01.

Because the disease spreads from EC to the hippocampus, we wanted to evaluate the effects of probiotic treatment on the hippocampus neural populations. The hippocampus is an extended structure and anterior and posterior hippocampal subfields might be differently affected during disease progression (Maruszack and Thuret, 2014). Therefore, we analyzed neural populations in the anterior as well as posterior hippocampus, using Bregma −2.0 as a reference mark. In the anterior hippocampus (−2.00 to −1.01 mm Bregma), we observed a significantly higher number of NeuN^+^ cells (Figure 5A) in ADP (37.43 ± 5.66%) compared to ADC mice (16.85 ± 2.57%, *p* = 0.007). Although trending downward, no significant changes in percentages of glia cell populations (GFAP^+^ and Iba1^+^) were observed between the two groups. As shown in Figure 5B, no differences in the percentage of positive neurons (NeuN^+^) were observed between ADC and ADP (22.08 ± 5.73% vs. 28.77 ± 6.35%) in the posterior hippocampus (−2.01 to −3.0 mm Bregma). Likewise, the percentages of astrocytes (GFAP^+^, 32.57 ± 2.62% vs. 27.26 ± 2.30%) and microglia cells (Iba1^+^, 33.73 ± 6.66% vs. 35.88 ± 4.16%) did not change between the non-treated and treated animals (Figure 5B).

**Figure 6:**
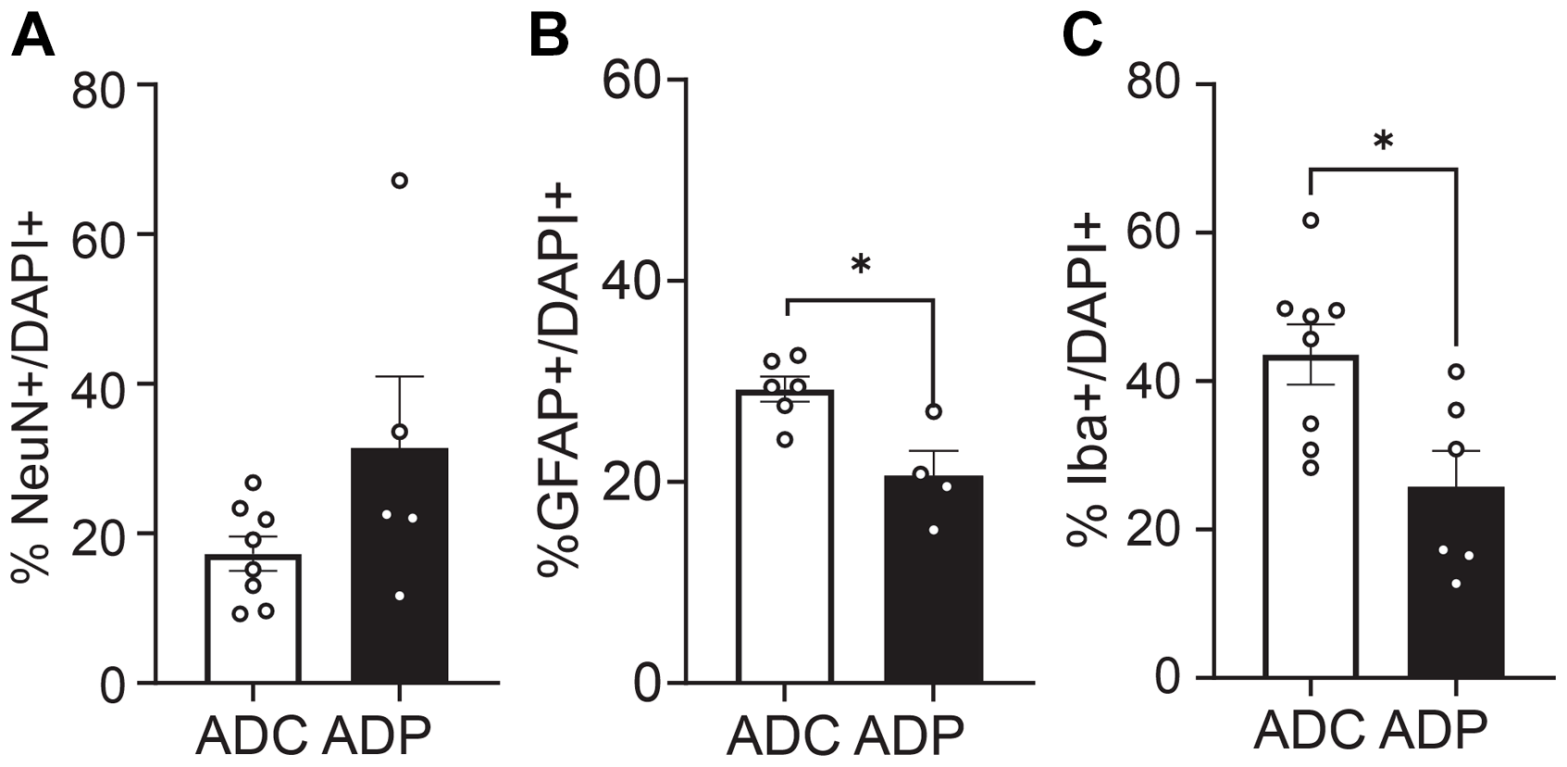
Neural cell populations in female mice were significantly affected by probiotics treatment in the anterior hippocampus. Percentages of **(A)** neurons (NeuN^+^), **(B)** astrocytes (GFAP^+^) and **(C)** microglia (Iba1^+^) were evaluated in 10 months old AD female mice. Given the small number of females in each group (*n* = 3 ADP and *n* = 4 ADC), each data point represents the average counts of 3 fields per section. All stained sections of each animal in a group are represented in the group. Data reported as mean percentage per group and analyzed by Mann–Whitney test. *p < 0.05.

However, since Alzheimer’s disease predominantly affects women, we were interested in the possible differential effects of probiotics on the two sexes. Further analysis showed that female mice were more affected by probiotic treatment than males. In the EC, the percentage of GFAP^+^ cells remained significantly lower in ADP females compared to ADC females (difference 3.69 ± 1.64%, *p* = 0.0296, data not shown). Importantly, in the anterior hippocampus (Figure 6) females exhibited significant differences in GFAP^+^ as well as Iba1^+^ cell populations. Elevated NeuN^+^/DAPI^+^ (17.28 ± 2.30% vs. 31.41 ± 9.59%, *p* = 0.171) and significantly decreased Iba1^+^/DAPI^+^ cell counts (43.49 ± 4.08% vs. 25.79 ± 4.83%, *p* = 0.043) were detected in ADP female mice. In addition, decreased GFAP^+^/DAPI^+^ cell counts were detected in ADP compared to ADC (20.64 ± 2.44% vs. 29.23 ± 1.25%), resulting in a significant difference (*p* = 0.019).

### 3.3 Probiotics affect Bacteroidetes abundance in 3xTg-AD mice

**Figure 7:**
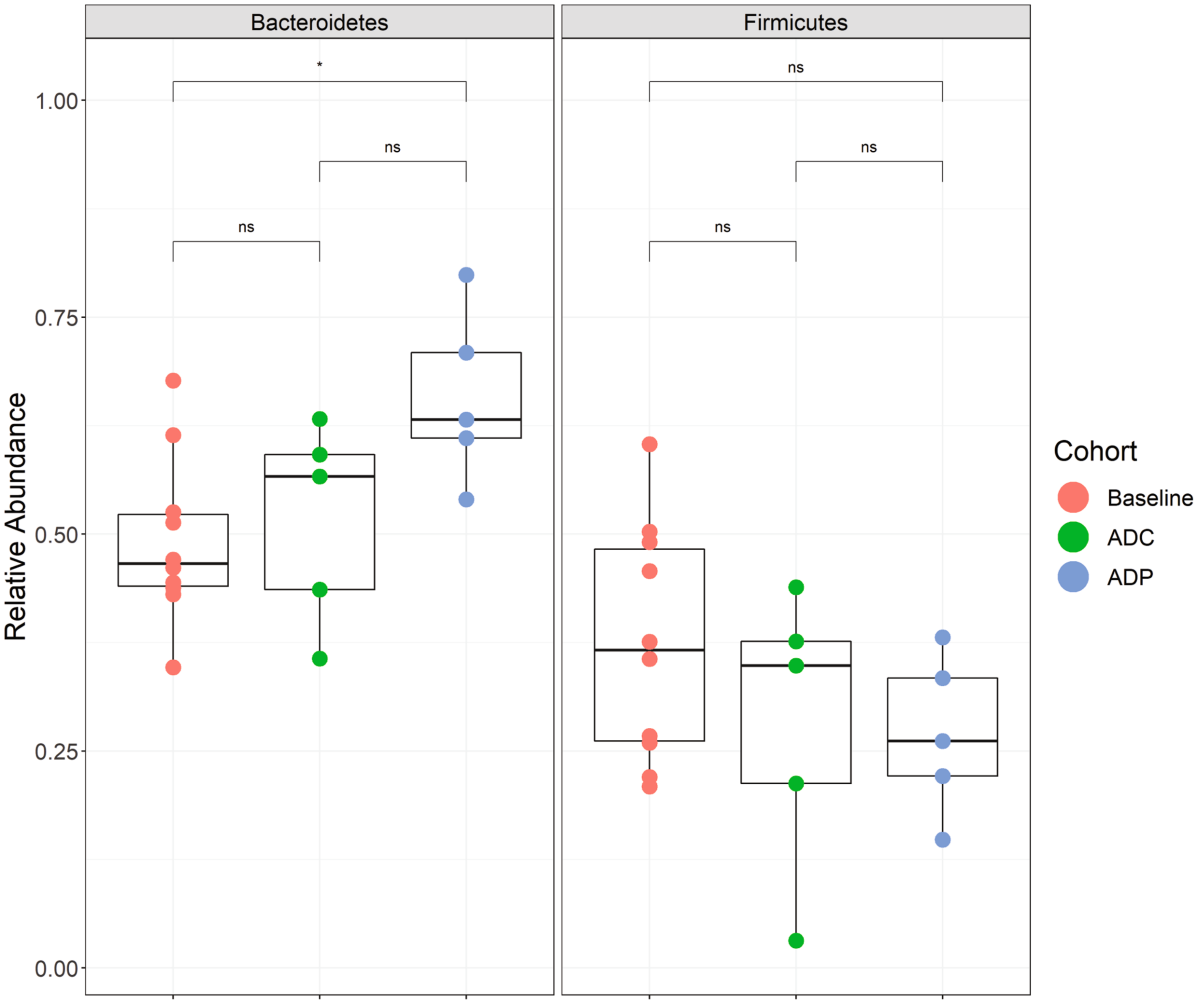
Relative abundance boxplots of Bacteroidetes and Firmicutes. Phylum-level relative abundances are plotted by group with baseline (red) indicating samples from all 10 mice before treatment (week 0), control (green) indicating the 5 mice that did not receive a probiotic supplement (week 12), and the probiotic (blue) group that received the probiotic treatment (week 12). A significant difference was observed when comparing the baseline samples to the probiotic samples for Bacteroidetes abundance (*p* < 0.05). Boxplots are presented in Tukey-style. The minimum sequencing depth for these samples was 14,112 reads.

We next wanted to determine if the neuroprotective effects of the probiotic treatment in AD mice correlated with a change in gut microbiota. Therefore, to assess the changes in the gut microbial composition of AD mice treated with probiotics, fecal microbiota were analyzed via the V4 region of the 16S rRNA gene. Since the V4 region of the 16S rRNA gene was used, we could only resolve the *Lactobacillus* species to the genus level. We did not observe any significant differences in beta- (Supplementary Figure S1) or alpha-diversity (Supplementary Figure S2) in AD mice receiving probiotics versus the AD control group. We also did not observe any significantly elevated bacterial groups when the ANCOM-BC test was performed on these data at all taxonomic levels (phylum through genus). However, we did observe that the probiotic treatment produced significant changes in one of the two dominant phyla, Firmicutes and Bacteroidetes, within the gut. The abundance of Bacteroidetes was significantly increased in the ADP mice after 12 weeks of receiving probiotics when compared to pre-treatment (adjusted *p* = 0.046) (Figure 7). These changes were not observed in the untreated AD mice (Figure 7).

## 4 Discussion

In the past decade, research has intensified around the role of the gut microbiota on human health, with an emphasis on brain health (Shreiner et al., 2015; Rinninella et al., 2019). In our current study, we supplemented the diet of 3xTg-AD mice with two *Lactobacillus* strains and evaluated their effects on AD disease progression. GM dysbiosis in the form of decreased microbial diversity, the presence or absence of specific genera, and shifts in the relative abundance of the two dominant phyla - Firmicutes and Bacteroidetes - have been linked to heightened AD pathology in clinical and preclinical studies (Brandscheid et al., 2017; Harach et al., 2017; Vogt et al., 2017; Zhuang et al., 2018; Sun et al., 2019; Chen et al., 2020; Hung et al., 2022). Efforts to correct gut dysbiosis through antibiotic treatment, fecal microbiota transplantation, and probiotic treatment have been shown to reduce AD pathology (Minter et al., 2016; Bonfili et al., 2017; Sun et al., 2019). Here, we did not observe any significant differences in microbial diversity or relative abundance at the genus level in AD mice receiving probiotics versus the AD control group, which could be due to the relatively small sample size in our study. However, we did detect a significant increase in the abundance of Bacteroidetes phylum in 10-month-old AD mice receiving *Lactobacillus*. The ratio of Firmicutes to Bacteroidetes has been used as an indicator of gut dysbiosis and variations are often correlated with, besides AD, diseases such as obesity and inflammatory bowel disease in humans. A decrease in Firmicutes*/*Bacteroidetes ratio or an increase in Bacteroidetes was correlated with health improvements in juvenile rodents (Stojanov et al., 2020). Nonetheless, there can be a great deal of variation in what is considered a ‘healthy’ Firmicutes to Bacteroidetes ratio between individuals and the ratio can change over time (Duncan et al., 2008; Mariat et al., 2009; Huttenhower et al., 2012). In AD mouse models, studies revealed that the relative abundances of Firmicutes and Bacteroidetes can change over time as well (Brandscheid et al., 2017; Harach et al., 2017). Numerous studies have investigated microbial gut composition in AD mouse models and reported varying results at the phylum level when comparing AD to wild-type mice. These studies have reported inconsistent changes in both Bacteroidetes and Firmicutes levels when compared to wild-type controls, suggesting that general disruptions in gut flora contribute to AD pathology rather than specific taxonamic groups (van Olst et al., 2021). Various extrinsic (e.g., diet) and intrinsic (e.g., genetic background, sex, and age) factors could potentially account for the discrepancies between these studies and further studies will be necessary to elucidate the impact of phyla ratios on brain health.

Here, 3xTg-AD mice benefited from the increase in Bacteroidetes as demonstrated by the positive effects observed in memory performance after 12 weeks of probiotic treatment. While AD mice on a regular diet demonstrated deteriorated memory performance compared to healthy mice, probiotic treatment helped AD mice recover speed and accuracy in the Barnes Maze (Figure 1). Interestingly, *Lactobacillus plantarum* KY1032 was previously shown to enhance spatial memory in an age-dependent rat model (Jeong et al., 2015), which is consistent with the cognitive data obtained in our current study. In addition, *L. curvatus* HY7601 and *L. plantarum* KY1032 were used to assess their potential benefit on mice receiving a high-fat diet (Choi et al., 2016) and obese humans (Mo et al., 2022). In addition to minimizing weight gain, the groups receiving *L. curvatus* HY7601 and *L. plantarum* KY1032, produced significantly higher levels of blood adiponectin, a metabolic regulator linked to cognitive performance and neuroinflammation control (*e.g.*, pro-inflammatory cytokines and activated microglia) (Polito et al., 2020). Although additional studies would need to be performed, it is intriguing to speculate that *L. plantarum* KY1032 and *L. curvatus* HY7601 could have modulated the GM and potentially led to elevated adiponectin levels, with the subsequent beneficial cognitive effects observed in our ADP group. Given the important interplay between metabolic disorders and cognitive decline, dietary interventions beneficiating both conditions would be invaluable (Kouvari et al., 2022).

Our hypothesis, supported by the current tissue analysis, is that GM changes induced by *Lactobacilli* would affect the brain, probably via modulation of circulating pro-inflammation markers secreted by locally reduced astrocytes and/or microglia. It is well established that taming neuroinflammation would positively impact the neuronal population (Chitnis and Weiner, 2017; Kaur et al., 2019; Huffels et al., 2022), resulting in healthier neuronal pathways (e.g., EC-Hippocampus), with subsequent improved memory performance. In our studies, 3xTg-AD mice on a regular diet showed consistently increased counts of glial cells in both the EC (Figures 3, 4) and the hippocampus (Figure 6), suggestive of hyperplasia and early stages of reactive gliosis. Probiotic supplementation strongly decreased astrocytes in the EC and, although not significant, microglia counts were also altered in ADC mice in the anterior hippocampus (Figure 5A). These findings support the idea that reactive gliosis starts in the EC, spreads to the hippocampus, and can be modulated by probiotic supplementation. By reducing glia reactivity, probiotics could alter neuronal death processes. Indeed, as glia counts were reduced in ADP compared to ADC, neurons were more abundant in the CA1/CA2 subfield of the anterior hippocampus (Figure 5A) in the 10-month-old 3xTg-ADP mice. Our two cohorts of AD mice gave similar results, although the limited size of the groups in each experiment might have limited the sensitivity of some of the comparisons performed here. It would be important to expand these results by characterizing structural changes in microglia and astrocytes to confirm their reactivity states.

Remarkably, these effects were exacerbated in a sex-dependent matter as the decreased microgliosis and neuronal atrophy were significantly more evident in the hippocampus of ADP females. Interestingly, female 3xTgAD mice might be more susceptible to neuroinflammation than males, with reported instances of higher cognitive decline. Previous studies have demonstrated that females fed a high-fat diet, which heightens inflammation, exhibited more spatial and acquisition memory deficits than males (Clinton et al., 2007; Blázquez et al., 2014; Gannon et al., 2022). Evidence suggests that microbiota composition shows sex-related differences (Elderman et al., 2018) that can also affect immunoresponses (Markle et al., 2013; Fischinger et al., 2019), and gut microbiota alterations are prominent during the aging process (Falony et al., 2016; DeJong et al., 2020). In fact, a recent study demonstrated that microbiome and cytokines profiles were influenced by aging in a sex-dependent manner (Webster et al., 2022). Given the interrelation of sex, age, immunity, and gut microbial composition, it is conceivable that the aging female mice in our study exhibited an exacerbated glia response (ADC group) that could be more responsive to probiotic-induced changes in gut composition (ADP group). Moreover, some studies suggest that 3xTg-AD female mice carry a higher load of Aβ than males (Carroll et al., 2010; Hu et al., 2023), while others did not (Clinton et al., 2007). Aβ load has been linked to inflammation, which should then result in elevated reactive gliosis in ADC females. Although we detected intracellular Aβ accumulation in the hippocampus of AD mice, no beneficial effects of probiotic supplementation were observed on Aβ load in our preliminary results (data not shown). However, our tissue analysis was performed at 10 months compared to 4–6 months of age (Hu et al., 2023), the early stages of Aβ deposition in this mouse model, and only on the hippocampal tissue and not cortical regions (Carroll et al., 2010). Therefore, it is conceivable that toxic protein aggregates had exceeded a critical accumulation point by 10 months of age in the ADP mice and modulation was not detectable. Given the limited number of male mice in our cohorts, we could not perform direct comparisons between sexes. However, it would be important to determine the state of circulating inflammatory markers in both sexes to support the speculation that probiotics could be more efficacious in females or more advanced stages of disease progression.

As neurodegenerative disorders increasingly affect an aging population, the identification of therapies to alleviate symptoms and improve patients’ life is crucial. Our current study demonstrates that probiotic supplementation can attenuate the neuropathology and cognitive decline associated with AD by affecting local glia populations and sparing neurons from atrophy. In comparison to traditional pharmaceutical treatments for AD, probiotic therapy would be inexpensive and easy to implement, while limiting detrimental side effects. Given our sex-dependent results, it is essential to highlight that the diversity of gut microbiota populations across individuals and sexes could allow for optimal personalized therapy. Only a few probiotics have been approved by the FDA (Bhadoria and Mahapatra, 2011), and none have been approved as a treatment or preventative for AD (Ayichew et al., 2017). Interestingly, the two probiotics used in our study were recently used in a clinical trial to treat microbial gut dysbiosis associated with obesity (Mo et al., 2022). Numerous studies have examined the potential impact of different probiotic strains, some of which have been used in various combinations and at varying doses. Since the microbiome is unique to everyone, personalized formulations may need to be tailored for individuals to maximize efficacy and limit potential adverse outcomes. However, to ensure the optimal composition (e.g., strains and dose) of probiotics before their use as a therapeutic, more work must be done teasing out the molecular mechanisms linking the gut microbiota and AD pathology. As exploring the gut microbiota leads to novel therapies for AD, our research aims to show that simple diet-based interventions can provide a viable therapeutic option for palliative neurodegenerative therapy.

## Data availability statement

The raw data supporting the conclusions of this article will be made available by the authors, without undue reservation.

## Ethics statement

The animal study was approved by Institutional Animal Care and Use Committee of the University of Hartford. The study was conducted in accordance with the local legislation and institutional requirements.

## Author contributions

DM: Formal analysis, Investigation, Writing – original draft. KM: Conceptualization, Formal analysis, Investigation, Methodology, Writing – original draft. MP: Conceptualization, Formal analysis, Investigation, Methodology, Writing – original draft. KN: Investigation, Writing – original draft. TT: Formal analysis, Investigation, Methodology, Writing – original draft. JG: Methodology, Resources, Writing – review & editing. AS: Conceptualization, Funding acquisition, Methodology, Resources, Writing – review & editing. PS: Conceptualization, Data curation, Formal analysis, Funding acquisition, Methodology, Resources, Writing – original draft, Writing – review & editing.

## Footnotes

^1^ basespace.illumina.com

^2^ https://docs.qiime2.org/2018.8/

^3^ https://github.com/qiime2/q2-taxa
