# Supplementary figures and images for: Slowing Alzheimer’s disease progression through probiotic supplementation
Source: Front Neurosci. 2024 Mar 6;18:1309075. doi: 10.3389/fnins.2024.1309075 (PMC10950931; doi:10.3389/fnins.2024.1309075)

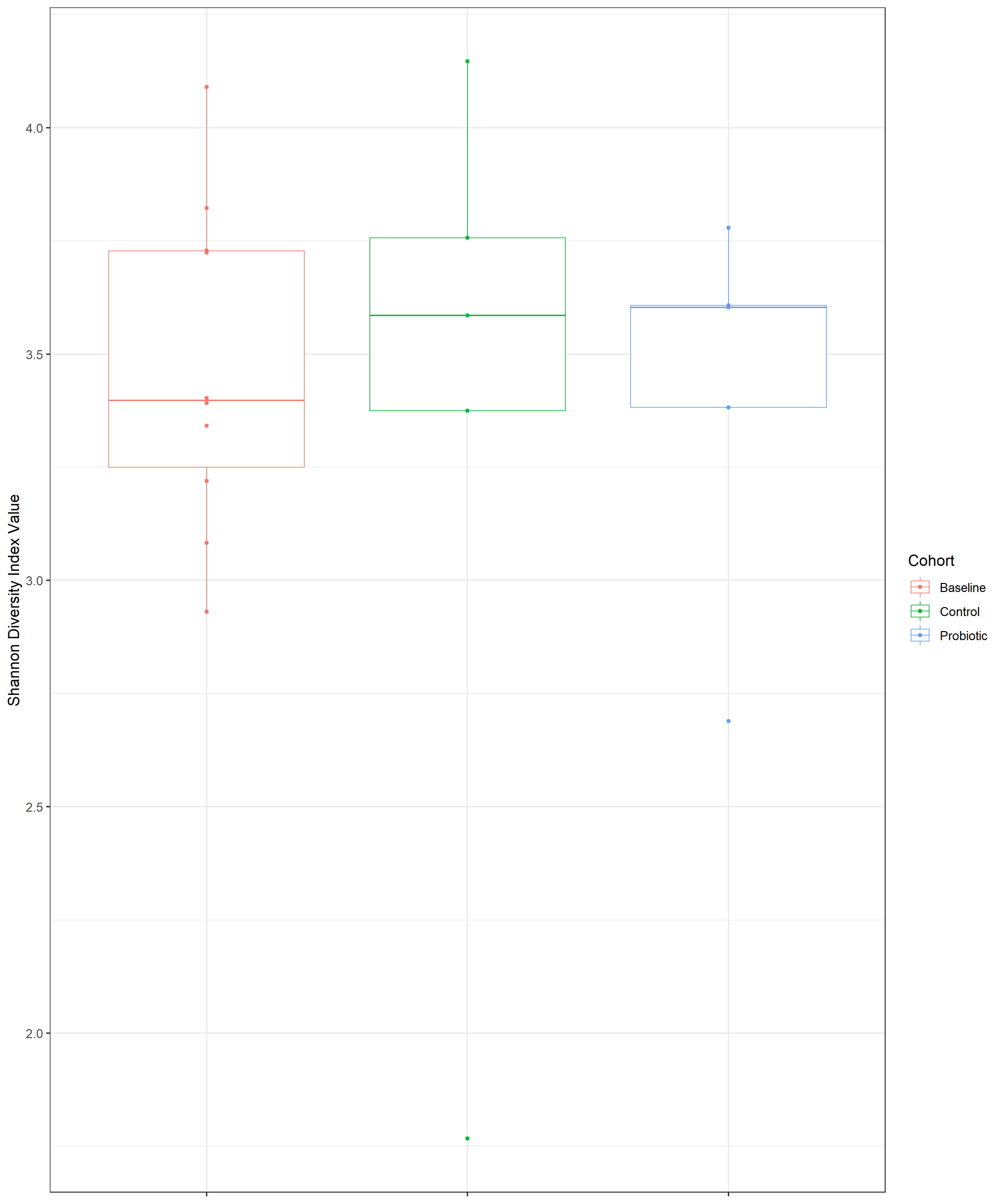

Supplement: Supplementary file 1 [file Data_Sheet_1.ZIP › Supple S2_alpha_div.tiff]

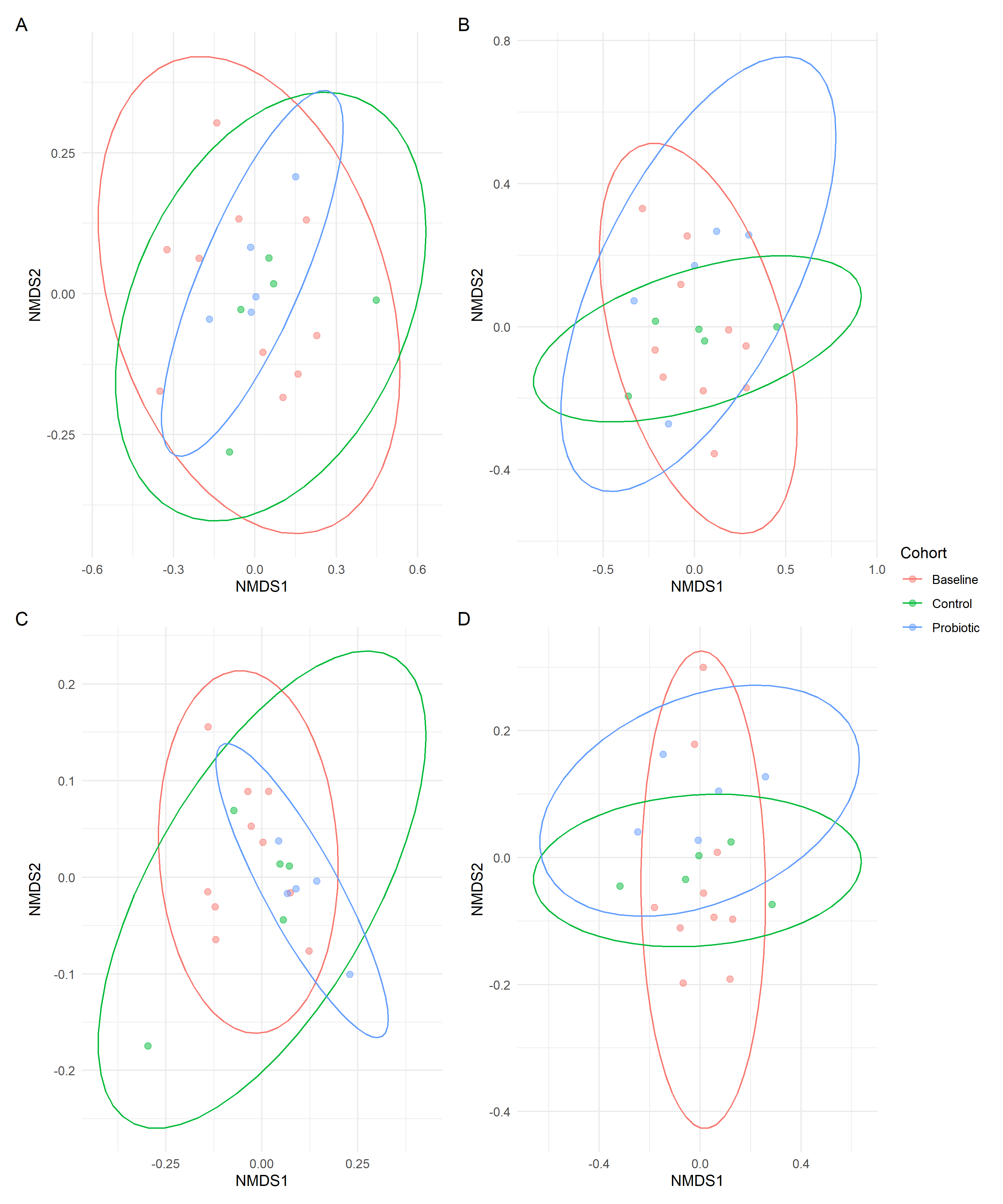

Supplement: Supplementary file 1 [file Data_Sheet_1.ZIP › Supple S1_beta_div.tiff]
